# Using Noninferiority Tests to Evaluate Telemedicine and E-Health Services: Systematic Review

**DOI:** 10.2196/jmir.2169

**Published:** 2012-09-28

**Authors:** Per Egil Kummervold, Jan-Are K Johnsen, Stein Olav Skrøvseth, Rolf Wynn

**Affiliations:** ^1^Northern Research InstituteTromsøNorway; ^2^University of Tromsø, Faculty of Health Sciences, Department of Clinical MedicineTromsøNorway; ^3^University Hospital of North Norway, Division of Addictions and Specialized Psychiatric ServicesTromsøNorway; ^4^University Hospital of North Norway, Norwegian Centre for Integrated Care and TelemedicineTromsøNorway

**Keywords:** noninferiority, non-inferiority, e-health, telemedicine

## Abstract

**Background:**

An increasing number of studies within the field of telemedicine and e-health are designed as noninferiority studies, aiming to show that the telemedicine/e-health solution is not inferior to the traditional way of treating patients.

**Objective:**

The objective is to review and sum up the status of noninferiority studies within this field, describing advantages and pitfalls of this approach.

**Methods:**

PubMed was searched according to defined criteria, and 16 relevant articles were identified from the period 2008-June 2011.

**Results:**

Most of the studies were related to the fields of psychiatry and emergency medicine, and most were published in journals relating to these fields or in general scientific or general medicine journals. All the studies claimed to be noninferiority studies, but 7 out of 16 tested for statistical differences as a proxy of noninferiority.

**Conclusions:**

The methodological quality of the studies varied. We discuss optimal procedures for future noninferiority studies within the field of telemedicine and e-health and situations in which this approach is most appropriate.

## Introduction

In the field of telemedicine and e-health, there is often a need to demonstrate that a new solution/application is equal in quality or efficacy of treatment to the traditional or established way of treating patients. Demonstrating superiority of the new solution in terms of quality or efficacy of treatment is not always necessary, as the telemedicine/e-health solution/application may have other types of advantages, including saved travel time or saved costs. Testing that the new solution is not inferior to a traditional counterpart may therefore seem to be sufficient in many cases. As would be expected from this line of reasoning, there has been an increase in published studies within the field of telemedicine and e-health, using a noninferiority design, ie, studies that aim to show that the new telemedical solution is not of a lower quality than the established way of treating patients.

In the present study, we performed a systematic review of the published literature and found 16 studies [1-16] within the field of telemedicine and e-health as commonly defined: “E-health is an emerging field in the intersection of medical informatics, public health and business, referring to health services and information delivered or enhanced through the Internet and related technologies” [17] and claiming to use noninferiority tests. We assessed the current status of the field and the strengths and weaknesses of the published studies.

The review aims to follow the criteria outlined in the PRISMA statement [18], but not all points are relevant since this is not a meta-analysis.

### Why is a Failed Test of Superiority not the Same as Noninferiority?

A good starting point for understanding what an insignificant result really means is by considering the famous quote by astronomer Carl Sagan: “Absence of evidence is not evidence of absence” [19].

Consider an experiment where we evaluate a video-based telemedicine service called T. We have decided to test whether this service is superior to a traditional clinical treatment called C. For simplicity we are looking at one single aspect, the patient’s blood sugar levels.

 We do a single sided *t *test of the mean blood sugar levels to check if T is superior to C, but we end up with a *P *value higher than .05. In other words, we have an insignificant result. Unfortunately, from a statistical point of view, this is nothing more than a failed test of superiority. It is not evidence that superiority does not exist. The only thing we are certain about is that our test was unable to prove any superiority.

The easiest way to understand this is that by reducing the number of participants, we are much more likely to get an insignificant result. It should be fairly obvious that a reduction in the number of participants is not making the groups more equal. It will result only in a study of lower quality and that is less able to detect if the new service is superior. 

Including more persons in the trial will increase the chance of detecting superiority (if it exists). However, whenever we end up with an insignificant result, we are still facing Sagan’s observation that the absence of evidence is not evidence of absence.

If the ultimate goal is to prove that service T is not inferior to service C, the only way of approaching this is to first define what we mean by “inferior”. Note that “inferiority” is an empirical definition. When comparing two groups in medical trials, we never end up with exactly the same results, and what margins we define should be based on clinical considerations of what are meaningful margins, not upon our ability to measure them.

In noninferiority trials, we therefore first define that a margin (M) below C is to be considered as noninferior. How to set this margin is discussed in “Methods”. We then go on to test if T really is superior to this margin.

## Methods

### Statistical Considerations

Testing for equivalence has become an essential statistical tool in the process of securing approval for new generic drugs [20]. Equivalence testing makes it possible to show that the generic drug is no different from the drug it is going to replace, without having to compare the new drug directly to placebo. Technically, a noninferiority test is nothing other than a one-sided equivalence test, requiring fewer participants to obtain the same power.

As described in the Introduction, there are multiple reasons a failed test of superiority is insufficient for concluding noninferiority, among them is sample that’s too small *(ie, lack of power*) or that the study is not able to detect a real world difference *(ie, lack of assay sensitivity*).

In order to demonstrate *noninferiority, *we need to define a margin for when the test group is worse than the control group. We call this the *noninferiority margin *and let M represent this value. If we let T represent the efficacy of the new test service and C represent the efficacy of the control, noninferiority can be expressed as: C − T < M. This is the alternate hypothesis in a noninferiority trial. The corresponding null hypothesis will be H_0 : _C − T ≥ M [21]. According to the CONSORT statement, a recommended way of performing a noninferiority test is constructing a two-sided 90% confidence interval (since α in principle may be different from 5%, the precise definition of the CI is 1-2α), and if the upper limit of the interval is less than M, the null hypothesis is rejected, ie, noninferiority is considered proven [22].

Setting the margin (M) must be done at the start of the trial, and in a clinical trial it should be related to what experts find clinically relevant. Wellek [20] stresses that the setting of M must be done after careful consideration in every project but mentions that everyday experience indicates that most people would consider a difference between C and T of 10% (strict) and 20% (liberal) to be of the same magnitude. This is also similar to what the FDA suggests as the threshold for establishing bioequivalence [23].

However, not only the difference between C and T is relevant for setting M. The margin must also be set in a way that a certain amount of the real effect of the active control over nontreatment/placebo (C-P) is conserved. Within biomedicine, it is discussed how small M could be in relation to C-P, and values ranging from 50-80% have been mentioned [21,24]. Setting M too small could lead to proving that the trial (T) is noninferior to the control (C), while at the same time not being clinically superior to nontreatment (P).

In an ordinary trial, a significant result does automatically prove the ability to detect a difference—typically called the trial’s *assay sensitivity*. A noninferiority trial does not have built-in assay sensitivity. Even if we get a significant result proving C − T < M, it is not proven that the two treatments have an effect. In fact, in a situation where our tools did not detect anything, we would also end up with C − T < M. In cases where it is impossible to include a placebo, assay sensitivity must be established drawing on historical data.

Summing up, the following factors are essential in noninferiority trials:

1. Finding a clinical relevant definition of M. M should be independent of factors like variance and sample size. While some have suggested that M could be in the range of 10-20% of C, this needs to be set individually for each project and must be done before the trial. It is not an error to clinically decide that M should be lower.

2. Making sure that M conserves the main effect between the active control and nontreatment. Values of M should be at least 50% of C-P.

3. Assuring assay sensitivity, either by including a placebo or by drawing on historical data.

Whether it is possible to find a formal determination of M and whether it is possible to prove assay sensitivity using historical data are both questions that are still discussed vigorously among statisticians [25].

### Search Strategy and Selection

The inclusion criteria are English-language articles that apply accepted definitions of telemedicine or e-health [17] and that use noninferiority tests as part of their methodology. The search terms are meant to reflect these criteria. A search in PubMed found 36 articles meeting the search criteria, which are given in Table 1.

**Figure 1:**
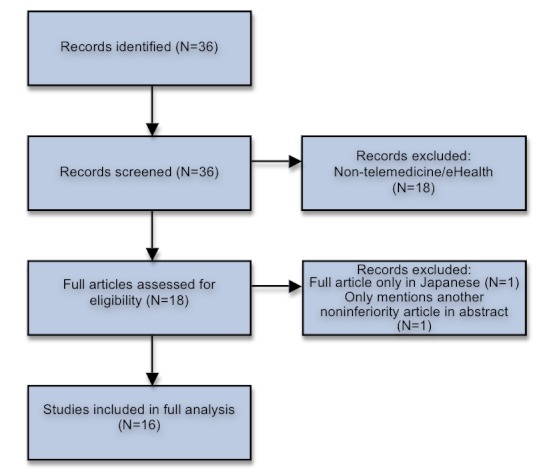
Strategy Flowchart.

Specific technological channels were included (eg, videoconference, Internet) in order to include articles within an intersection of fields that is not clearly defined as telemedicine or e-health in the article’s title or abstract. After the search, articles were manually scanned to exclude articles not fullfilling the inclusion criterias. Eighteen articles were excluded because they were clearly unrelated to telemedicine or e-health (in most cases this was caused by abstracts with the words “video” or “Internet”). One additional article was excluded because the main article was available only in Japanese, and another article was excluded since it referred to other noninferiority trials only in the abstract. This left 16 articles for further analysis (Table 2). The strategy is outlined in Figure 1.

Of the included articles, three were from 2008, three from 2009, five from 2010, and five from 2011 (until June 2011). No articles meeting the inclusion criteria were published prior to 2008.

**Table 1:** Description of search criteria.

**Search Criteria**
(noninferior OR noninferiority OR non-inferiority OR ("non inferior") OR ("not inferior")) AND (telemedicine[Title/Abstract] OR videoconference[Title/Abstract] OR video[Title/Abstract] OR videoconferencing[Title/Abstract] OR online[Title/Abstract] OR Internet[Title/Abstract] OR ehealth[Title/Abstract] OR e-health[Title/Abstract])

### Review Process

In the review of the articles, two reviewers (Authors 1 and 2) identified how the noninferiority margin was set and the reason that was provided for setting it. They also noted whether an actual noninferiority test was performed or if it was a test for difference. Finally, they registered how assay sensitivity was assured.

## Results

Six of the included articles dealt with matters related to psychiatric treatment (post-traumatic stress disorder, generalized anxiety disorder, depression), four of the articles dealt with medical procedures particularly relevant to emergency medicine (vascular access, defibrillation, advanced life support), one was within the field of urology, one within rehabilitation after surgery, one within endocrinology, one within hematology, and two within medical communication studies. With regard to where the papers were published, only one was published in a telemedicine journal, five were published in emergency medicine journals, two in a psychiatric journal, one in an orthopedic surgery journal, one in an endocrinology journal, and six in general scientific or general medical journals.

### The Setting the Noninferiority Margin

Various ways of defining the inferiority margin were used in the 16 articles reviewed (Table 2). In two articles [2,6], the inferiority margin was set to of 10% deviance from the main effect. In four articles [5,8,9,14], it was related to absolute values on validated questionnaires. In one of these articles [5], Cohen’s *d *= 0.5 was used for setting the inferiority margin for some of the measures. Cohen’s *d *was also used in another article [3], but here the margin was set to 0.2.

In one article [1], the margin was defined as 0.15SD. Another article [10] defined the noninferiority margin as a 10 percentage points difference between the proportions in the two groups.

Two studies referred to the lower bound of the confidence interval for the scores of the reference group. One of them [16] used the 90% confidence interval; the other [4] used a 95% confidence interval. In one article [11], the inferiority margin was set to RR = 0.95.

In the four remaining studies [7,12,13,15], the authors made no attempt to set an inferiority margin.

### Reasons Given for Setting a Specific Margin

Five articles [2,5,8,9,14] referred to expert consultations or clinical relevance as the main source for setting the margin, while two of them [2,9] also stated that this value was similar to the value set in prior studies.

In one article [6], it was argued that 10% is a typical value in medical trials. One article [1] stated that setting of the noninferiority margin was guided by Cohen’s [26] conventional criterion for small, medium, and large effect sizes.

One article [3] simply stated that the margin was defined as being relevant, while four articles [4,10,11,16] did not provide a reason.

In the remaining articles [7,12,13,15], the authors did not set a specific margin.

### Testing for Inferiority

Another question is whether a noninferiority test was actually performed, ie, that it was tested that the target effect was larger than the noninferiority margin. This could be accomplished either by checking whether the entire confidence interval for the means difference was above the noninferiority margin or by calculating a *P *value.

Nine of the articles [1,3,5,6,8-11,14] involved tests for noninferiority against the noninferiority margin. All except [3] found a significant result.

Seven of the articles [2,4,7,12,13,15,16] tested for differences instead. When this did not provide a significant result, they claimed noninferiority.

### Ensuring Assay Sensitivity

Four of the studies [2,13-15] had a pre-post design and were able to detect a significant difference between the start and end scores. This means that the studies had assay sensitivity. In some studies [1,5,6-9,16], one of several previously validated questionnaires was used—Patient Assessment of Communication during Telemedicine (PACT) [1], Liebowitz Social Anxiety Scale (LSAS) [5], Rapid HIV Pretest Information Comprehension [6], Clinician Administered PSTS Scale (CAPS) [7], State Trait Anger Expression Inventory-2 (STAXI-2) [8], Clinician Administered PSTS Scale (CAPS) [9], Novaco Anger Scale total score (NAS-T) [9], and AHA PALS Core Case Testing Checklist [16]. Since these questionnaires had previously shown significant results, it might be argued that this ensures assay sensitivity. It can be argued that [4] is in the same category, since measuring number of days in the therapeutic range is typically used when evaluating anticoagulants in other studies.

In [3], the authors included a noninferiority test but did not get a significant result. A difference test would show a significantly worse outcome for the treatment group, and the study does therefore, albeit indirectly, have assay sensitivity. In [10-12], we were not able to identify attempts at proving assay sensitivity.

**Table 2:** Articles included in review.

**Reference**	**Margin**	**Reasoning**	**Test**
Agha et al, 2009 [1]	0.15 SD	Guided by Cohen	Noninferiority
Chenkin et al, 2008 [2]	10% from mean	Clinically + prior studies	Difference
de Vries et al, 2010 [3]	Cohen’s *d *= 0.2	Defined	Noninferiority
Harper & Pollock, 2011 [4]	Unclear: a) Within 5%, b) Lower bound 95% CI	No reason given	Difference
Hedman et al, 2011 [5]	Absolute value + Cohen’s *d *= 0.5	Clinically + prior studies	Noninferiority
Merchant et al, 2009 [6]	10% from mean	Typical in medical trials	Noninferiority
Morland et al, 2011 [7]	Not set	Not relevant	Difference
Morland et al, 2010 [8]	Absolute value	Clinically	Noninferiority
Morland et al, 2009 [9]	Absolute value	Clinically + prior studies	Noninferiority
Mpotos et al, 2011 [10]	10 percentage points difference in proportions	No reason given	Noninferiority
Munger et al, 2008 [11]	RR=0.95	No reason given	Noninferiority
Péres-Ferre et al, 2010 [12]	Not set	No reason given	Difference
Robinson et al, 2010 [13]	Not set	Not relevant	Difference
Russell et al, 2011 [14]	Absolute value	Clinically	Noninferiority
Titov et al, 2010 [15]	Not set	Not relevant	Difference
Weeks & Molsberry, 2008 [16]	Lower bound 90% CI	No reason given	Difference

## Discussion

As the results show, there are considerable variations in the way the noninferiority trials are performed. The 16 included articles should encompass the majority of the studies that claim to be noninferiority trials within the field of telemedicine and e-health, but a few that have not been indexed in PubMed might have been missed. While the study method seems to be growing in popularity, it is still in its infancy. Most current use of noninferiority trials is within biomedicine, and there are, as we have shown, only a few examples of use within telemedicine and e-health. While noninferiority trials within biomedicine can serve as an inspiration, differences between the fields make it difficult to copy the approaches used in biomedical trials. Below, we discuss some of the central elements of noninferiority trials and how they can be applied to studies within telemedicine and e-health.

### Setting the Margin

To prove that something is equal, or not inferior, we need to define what we mean by equality or noninferiority. This is mainly a clinical issue that primarily should be assessed by experts within the field. Some very rough guidelines have been referred to, and values within 10-20% appear to be considered fairly equal in the literature. What is clinically relevant cannot be decided by this value alone. In some cases, a 10% difference can have enormous impact, while in other cases this value is clinically irrelevant. Only five of the articles included in our review referred to the concept of clinical relevance.

There are other guidelines stating that the margin should be set so that a majority of the effect between the control (C) and the nontreatment (P) should be preserved. In trials where the nontreatment group is not included, the researcher will have to estimate the effect of C-P based on previous trials. This is not a luxury that many telemedicine/e-health trials have.

### Proving Noninferiority

When performing a traditional hypothesis test, a *P *value higher than the significance level does not provide evidence that the null hypothesis is true. It simply means that the evidence is not strong enough to reject the null hypothesis with sufficient confidence. Indeed, it is possible that a study that results in a *P *value above the significance level will be a positive contribution to a future meta-analysis in proving that there is a difference. The most surprising result of our review is that almost half (7 of 16) of the articles seem to disregard this fact. They actually performed tests of difference, and their main argument for noninferiority was that the difference test gave an insignificant result.

### Assay Sensitivity

One of the main driving forces in the popularity of noninferiority and equality testing within biomedicine is that it enables doing evidence-based medicine without including a nontreatment group. In some cases it might be ethically unacceptable to introduce a placebo. In other cases, this is primarily a question of cost saving. It might be fair to say that the increasing use of noninferiority and equality testing is related to the growth of so-called explanatory or pragmatic trials, where the main question is not whether a treatment is effective but whether the treatment is worthwhile using in a clinical setting [27,28]. 

Ideally, assay sensitivity should be proven by a previous trial or a meta-analysis of multiple previous trials. It is difficult to replicate studies in this fashion within the field of telemedicine/e-health, and none of the studies examined in our review did this. However, 7 of the 16 studies did use previously validated questionnaires, an alternative that in many cases actually may be sufficient.

There were also four studies that included a placebo/no treatment. For simply proving assay sensitivity, this is definitely sufficient. It is, however, a bit contrary to the original purpose of equality and noninferiority tests, which is to be able to do without a placebo/no treatment group.

The review did also identify three studies where there were no explicit indications in the articles that assay sensitivity had been established. The authors might, however, have carried out such procedures without reporting it.

### Recommendations

As the analysis shows, the fundamentals of noninferiority testing can be daunting to use in practice, especially for authors that are new to this type of analysis. We recommend that authors pay close attention to the extended CONSORT guidelines for noninferiority testing to the extent that they are applicable for the study in question [22]. We believe that one of the articles by Morland et al [8] provides a good example of how a noninferiority article can be performed and reported. Morland et al [9] also provide a more detailed methodological discussion concerning noninferiority trial design.

Performing a noninferiority study requires, as with any choice of statistical analysis, strict adherence to protocol to avoid fishing for positive results, which will dramatically affect the probability of type II errors. In particular, the noninferiority margin must be set before the study starts. Setting the margin after investigating the data means the investigator essentially can obtain any result wanted. Similarly, if an investigator performs a standard superiority trial and finds a nonsignificant result, the study should never be transformed into a noninferiority study. The intent of determining noninferiority must be clear from the outset.

When there is sparse evidence for assay sensitivity, such as if there are few studies to base the analysis on, noninferiority testing may not be the best option. Assay sensitivity is essential for doing a proper noninferiority study since without it, the study could end up proving that the intervention is no worse than doing nothing (ie, does no harm). In such settings, it should be considered if another type of design is more appropriate, eg, an economic evaluation.

### Conclusions

Noninferiority testing clearly has a place within telemedicine and e-health. It is, however, always a much more daunting task to prove that something (like a difference) does not exist than to prove that it does exist. As we have discussed in our review, noninferiority trials are not a magic shortcut to solving this fundamental challenge.

While several of the trials included in this review are of a high quality, the review also brings to light an apparent lack of awareness of the pitfalls of performing noninferiority trials. We recommend more stringent adherence to the basic principles of noninferiority testing. We have discussed some points that should be given specific attention, including the importance of not mistaking a failed difference test for proof of noninferiority and the importance of setting a clinically relevant noninferiority margin. 
